# Evolution of Primary Hemostasis in Early Vertebrates

**DOI:** 10.1371/journal.pone.0008403

**Published:** 2009-12-23

**Authors:** Seongcheol Kim, Maira Carrillo, Vrinda Kulkarni, Pudur Jagadeeswaran

**Affiliations:** Department of Biological Sciences, University of North Texas, Denton, Texas, United States of America; Texas A&M University, United States of America

## Abstract

Hemostasis is a defense mechanism which protects the organism in the event of injury to stop bleeding. Recently, we established that all the known major mammalian hemostatic factors are conserved in early vertebrates. However, since their highly vascularized gills experience high blood pressure and are exposed to the environment, even very small injuries could be fatal to fish. Since trypsins are forerunners for coagulation proteases and are expressed by many extrapancreatic cells such as endothelial cells and epithelial cells, we hypothesized that trypsin or trypsin-like proteases from gill epithelial cells may protect these animals from gill bleeding following injuries. In this paper we identified the release of three different trypsins from fish gills into water under stress or injury, which have tenfold greater serine protease activity compared to bovine trypsin. We found that these trypsins activate the thrombocytes and protect the fish from gill bleeding. We found 27 protease-activated receptors (PARs) by analyzing zebrafish genome and classified them into five groups, based on tethering peptides, and two families, PAR1 and PAR2, based on homologies. We also found a canonical member of PAR2 family, PAR2-21A which is activated more readily by trypsin, and PAR2-21A tethering peptide stops gill bleeding just as trypsin. This finding provides evidence that trypsin cleaves a PAR2 member on thrombocyte surface. In conclusion, we believe that the gills are evolutionarily selected to produce trypsin to activate PAR2 on thrombocyte surface and protect the gills from bleeding. We also speculate that trypsin may also protect the fish from bleeding from other body injuries due to quick contact with the thrombocytes. Thus, this finding provides evidence for the role of trypsins in primary hemostasis in early vertebrates.

## Introduction

Hemostasis is a defense mechanism that evolved to protect the organism in the event of injury to stop bleeding [1]. In mammals, when a vessel wall is ruptured, the tissue factor on the surface of the cells in the subendothelial matrix binds to the preexisting factor VIIa and initiates coagulation by cleaving factor X to Xa, which then cleaves prothrombin to generate minuscule amounts of thrombin. This thrombin cleaves factor XI to XIa. XIa then cleaves factor IX to IXa, which along with cofactor VIIIa, cleaves larger amounts of factor X to Xa, which then generates explosive amounts of thrombin from prothrombin. Interestingly, these reactions occur on the surface of platelets that are anchored to the subendothelial matrix via collagen and vWF, which themselves activate the platelets along with the newly generated thrombin. These events initiate the secretion of ADP, thromboxane and serotonin, which activate and amplify the aggregation of platelets.

Recently, we established that almost all the known major mammalian hemostatic factors are conserved in early vertebrates [2]. However, since the highly vascularized gills which experience high blood pressure are exposed to the environment, even very small injuries could be fatal to fish. Therefore, these vertebrates must have evolved mechanisms by which they can respond to an injury or even stress that could cause bleeding in gills. We do not know whether there are novel mechanisms for activation of thrombocytes to enhance hemostasis in fish gills.

Trypsins which are mainly produced in pancreas and are involved in digestion are the evolutionary forerunners for coagulation proteases [3], [4]. These are also expressed by many extrapancreatic cells such as endothelial cells, epithelial cells, nervous system and tumor cells [5]. Trypsin, while used mainly for cleaving extracellular proteins in digestion, also cleaves the membrane proteins called protease-activated receptors (PARs) similar to the way thrombin digests PARs on platelet surface and initiates signaling cascade in enterocytes that are in contact with trypsin [6], [7]. However, nothing is known about the function of extrapancreatic trypsins and the possible cells that they will be activating although several studies are suggestive of their role. We hypothesized that trypsin or trypsin-like proteases from epithelial cells from organs such as gills may protect these animals from gill bleeding following injuries. In this paper we identified trypsin in fish gills and its release under stress or injury into water. We also found that the trypsin released around the gills activates the thrombocytes and plays a role in primary hemostasis.

## Materials/Methods

All experiments described below were approved by the Institutional Animal Care and Use Committee.

### Chromogenic Assay

60 zebrafish (Ekkwell, Gibsonton, FL) maintained under standard conditions were kept in 150 ml distilled water for 20 min. The fish were then removed and water was centrifuged at 7,000 g for 10 min in a Sorvall centrifuge to remove the feces and other particulates. This particulate free water was then lyophilized and the dry pellet was dissolved in 200 µL water. This lyophilized zebrafish water contains proteins and this concentrated protein solution is called ZW. The protein concentration was estimated by BioRad Bradford assay kit (Bio-Rad Laboratories Inc., Hercules, CA) using 10 µL of this ZW. S-2238 activity assay was performed using 5 µL of ZW in a final assay volume of 200 µL containing 500 µM S-2238 in 50 mM Tris-HCl pH 7.2 ( S-2238 is a thrombin substrate but trypsin and other serine proteases also cleave this chromogenic substrate and since it was readily available in our laboratory we used this substrate). The reaction was incubated for 15 min and yellow color was measured at 405 nm in a 96-well kinetic microplate reader (Molecular Device Company, Sunnyvale, CA). For in-gel chromogenic assay, the lane containing the fish protein was sliced and kept in a small tray and the S-2238 substrate was layered on the gel and incubated for 15 min. The gel was photographed using a Nikon E995 CoolPix digital camera. Gill, mouth and nasal washes each was carried out on 200 fishes by using 10 µL of water and pipetting up and down three times in these cavities and then samples were pooled, lyophilized and used in the above chromogenic assays. Animal care was in accordance with institutional guidelines. Kinetic analysis was performed by standard methods.

### Analysis of Proteins

The water proteins were resolved on 5–20% premade polyacrylamide denaturing gel gradient containing 1% SDS (Bio-Rad Laboratories Inc., Hercules, CA). The samples were boiled just before loading. The gel was washed to remove SDS and renatured for 1 hr with renaturation buffer (Bio-Rad Laboratories Inc., Hercules, CA) before performing the in-gel chromogenic assay as described above. The gel band that showed positive activity was sent to the core facility at University of Texas-Southwestern Medical Center at Dallas for MALDI-TOF sequencing. For Western blot, ZW was resolved on a 5–20% SDS-poly acrylamide gradient gel and then transferred to a nitrocellulose membrane, which was probed with polyclonal rabbit antisera against human trypsin (PRAHT) and followed by probing with alkaline phosphatase conjugated (AP) goat anti-rabbit IgG(Fc). The probed bands were visualized using the AP substrate BCIP/NBT.

### Functional Evaluation Assays

Thrombocyte functions were assayed by whole blood aggregation using a plate tilt assay [8]. Annexin V binding assays were performed as described earlier [9]. For gill bleeding assay, fish were placed in 15 ml of 10 mM NaOH solution to induce bleeding, for 1.5 min with distilled water, 70 ng of ZW, 70 ng of ZW with 140 ng of PRAHT and 70 ng of ZW with 140 ng of control IgG. The images taken of the zebrafish were photographed using a Nikon E995 CoolPix digital camera. The red color pixels were counted as an index of the area indicating the extent of bleeding using Adobe photoshop software version 7.0. For calcium release assay, Xenopus oocytes were microinjected with 50 nl of PARs mRNA cloned in pSP64T in 10 mM HEPES (pH 7.0) [10]. The oocytes were retained in 16°C for 48 hrs, and then incubated with ^45^Ca^2+^. The oocytes were then washed and a scintillation count was taken using LC-6000 liquid scintillation counter (Beckman Coulter, Inc.) at 10 min intervals to determine amount of calcium released. After 20 min, calcium release was induced using 5 ng ZW as the agonist. The released ^45^Ca^2+^ from oocytes was measured again at 10 min intervals.

### Expression and Activity Analysis of Fish Trypsins

Zebrafish trypsin full length cDNAs were generated by RT-PCR and these cDNAs were cloned into the *E.coli* expression vector pMALc2e and after affinity purification and enterokinase cleavage, trypsin activity was estimated.

### Immunohistochemistry

Adult zebrafish was fixed in a fixative, sectioned into 5 micron thick slices and probed with the anti rabbit antibodies raised against human trypsin. Secondary antibodies raised against rabbit with conjugated FITC were used. The images were taken by using Nikon Eclipse 80 microscope with constant exposure times and also by using CSU-10 YoKogawa confocal scanner (McBain Instruments, Simi Valley, CA) coupled to Zeiss 200 M microscope (Carl Zeiss, Inc. Thornwood, NY).

## Results/Discussion

### Identification Serine Protease in Zebrafish Water

**Figure 1:**
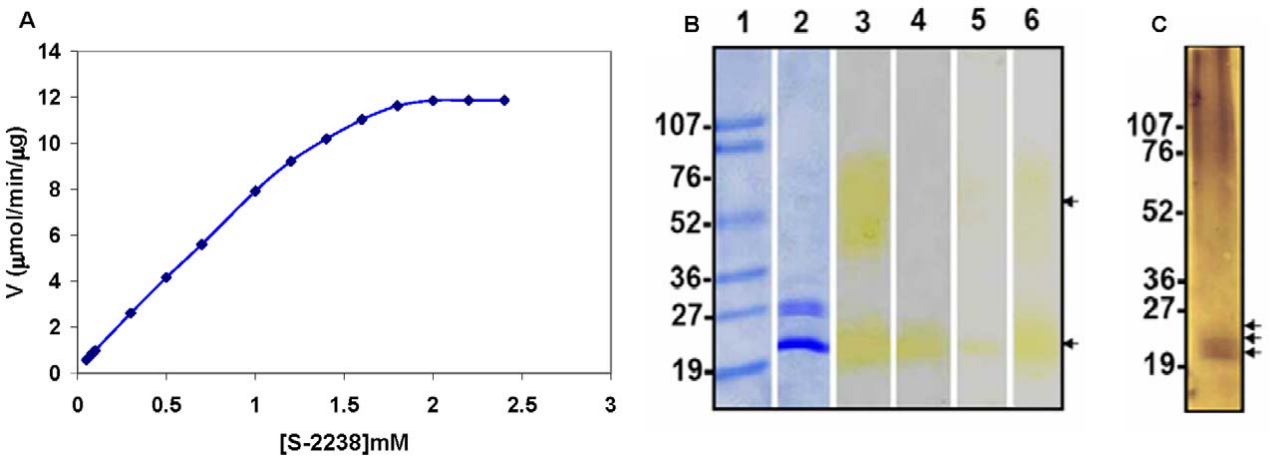
Protease activity in zebrafish water samples. (A) Michaelis-Menten plot with velocity as a function of [S-2238]. Each sample was maintained for 30 min prior to mixing with substrate under the specified conditions. Standard error bars (±0.07) were removed to improve clarity of each graph. (B) The proteins secreted by zebrafish were separated on a 5–20% Tris-glycine SDS-polyacrylamide gradient gel. The samples loaded in the lanes are as follows: 1, molecular weight marker (numbers on the left side indicate the size of the band in kDa); 2, 3, ZW; 4, zebrafish mouth wash; 5, zebrafish nose wash; and 6, zebrafish gill wash. Lanes 1 and 2 were stained with Coomassie blue dye. Other lanes were stained using 10 mM S-2238 following renaturation. Arrows indicate protease activity. (C) Western blot analysis of ZW. Arrows indicate trypsins. The numbers on the left side indicate the size of the molecular weight markers in kDa.

We hypothesized that trypsin or trypsin-like proteases from epithelial cells of organs such as gills may protect these animals from gill bleeding following injuries. We further hypothesized that if this activity is found in gills it should be secreted from gills and should be found in water. To determine the presence of proteases in fish water we used the serine protease substrate S-2238 [11]. Zebrafish were housed in fresh water for 20 min, and then the water was collected, centrifuged to remove debris, lyophilized, and dissolved in a minimal volume. This zebrafish water sample contained sufficient enzyme to convert 0.5 ng S-2238/min/gram of fish using cleavage by human thrombin as the positive control. This assay yielded reproducible Michaelis-Menten kinetics with a Km of 0.83 mM and a Vmax of 13 µmol/mM/µg of ZW (Figure 1A). The sample exhibited a broad range of pH and temperature dependence. Interestingly, after boiling and slowly cooling the ZW, the enzymatic activity was almost completely recovered. In addition, the sample was stable in 2 M urea; was not altered in the presence of CaCl_2_; retained partial activity in 1% SDS; was not inhibited by EDTA (Figure S1), benzamidine, or PMSF; and was inhibited by leupeptin and antipain.

### Characterization of Protease in ZW

To identify the protease that yielded SCA, the sample was run on an SDS denaturing gel, and then the gel was layered with S-2238 following renaturation in order to detect bands containing substrate cleavage activity. Two bands were identified: one at 55–75 kDa appearing as a smear and one at 25 kDa (Figure 1B). These bands were cut out, proteins eluted and analyzed by MALDI-TOF mass spectrometric sequencing. The 25 kDa band revealed trypsin peptides corresponding to three different trypsins (Table S1). In addition, superoxide dismutase and zinc metalloprotease peptides were detected in this band. Western blotting with polyclonal rabbit antisera against human trypsin (PRAHT) revealed three bands corresponding to three trypsins in this band supporting the data obtained by MALDI-TOF analysis (Figure 1C). Thus, the SCA resulting from three trypsins is consistent with the observed multiple peaks for the pH optima (Figure S1). Also, these results demonstrated that PRAHT is specific and does not cross react with any other proteases. The 55–75 kDa band contained the serpins antitrypsin and serpin a1, proteases such as trypsins and legumain-like protease, and other proteins unrelated to SCA (Table S2).

### Expression of Protease in Tissues

**Figure 2:**
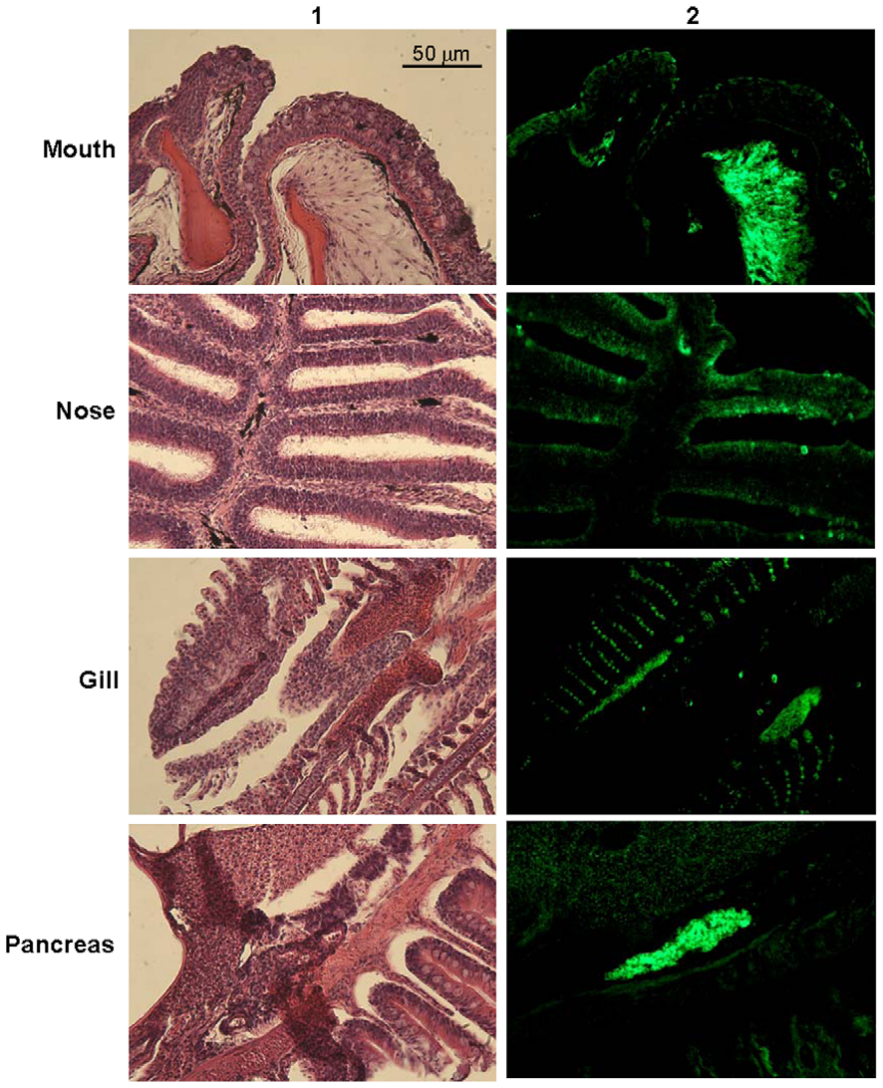
Immunohistochemistry of trypsin in zebrafish. Zebrafish were dissected and sections of mouth, nose, gill, and pancreas were stained with H&E (1) and subjected to immunohistochemistry analysis using polyclonal rabbit antisera against human trypsin (PRAHT) (2). Alexa goat anti-rabbit IgG 488 antisera were used as the secondary antibody. Non-immunized rabbit IgG was used in control experiments (images not shown). 20X lens was used.

**Figure 3:**
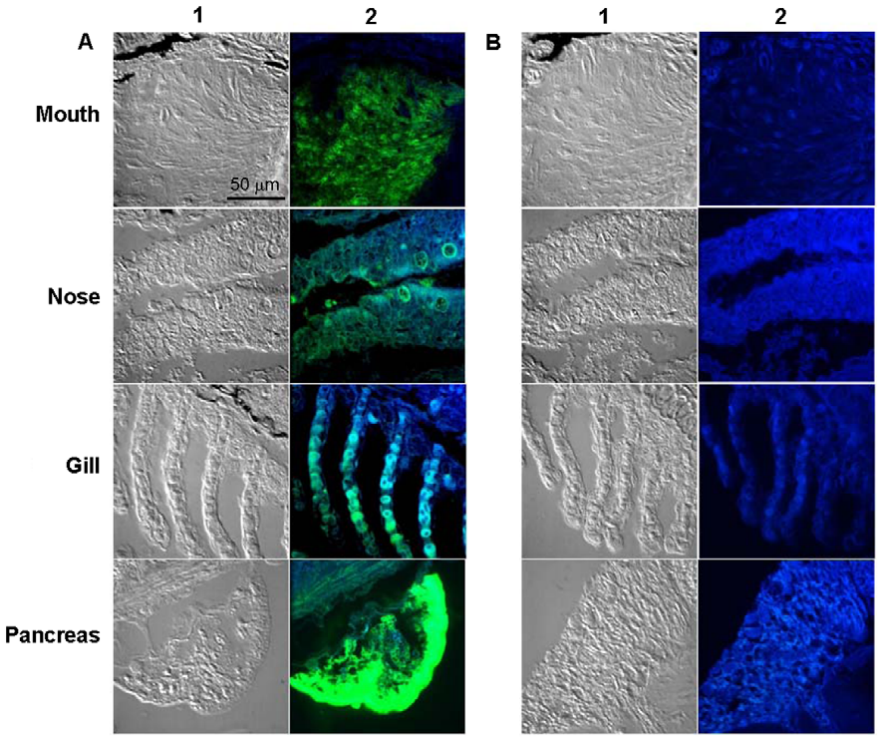
Detection of trypsin by confocal microscopy. Confocal DIC images (a, 60X and b, 100X) of zebrafish sections of mouth, nose, gill and pancreas stained by immunohistochemistry using primary antibody (a) PRAHT, (b) Non-immunized rabbit IgG. Alexa Fluor 488 goat anti-rabbit IgG was used as secondary antibody. We used two channel image acquisition to examine the expression of trypsins that were probed with Alexa Fluor. A 488 nm and a 405 nm laser were used to excite the Alexa Fluor (Green) and autofluorescence (Blue), respectively. Columns 1 and 2 show brightfield and fluorescence images respectively.

To test from where the trypsin activity is derived we collected water after immersing the fish head alone and also immersing the rest of the body. The water from immersing the head generated activity whereas the water collected from rest of the body did not produce trypsins ruling out the skin as a source for trypsin. Subsequently the gill wash, nasal wash, and mouth wash samples were analyzed which yielded trypsin activity corresponding to the 25 kDa band, whereas only the samples from the gill wash corresponded to the larger band. Since water collected after immersing the fish head alone and then immersing the rest of the body did not produce trypsins, the skin was not implicated as a source of these enzymes. In addition, RT-PCR demonstrated that trypsin mRNA is synthesized in gills, nose, and mouth tissues (Figure S2). Immunoreactive cells were also detected in the gills and nasal epithelium (Figure 2). Counterstaining with histamine antibodies revealed that the trypsin-producing cells are not mast cells [12] (data not shown). Skin did not show any immunoreactive cells (data not shown). Confocal microscopy also revealed positive staining in all the tissues as was observed by the standard microsopy (Figure 3).

### Stress Induced Synthesis of Protease Activity

**Figure 4:**
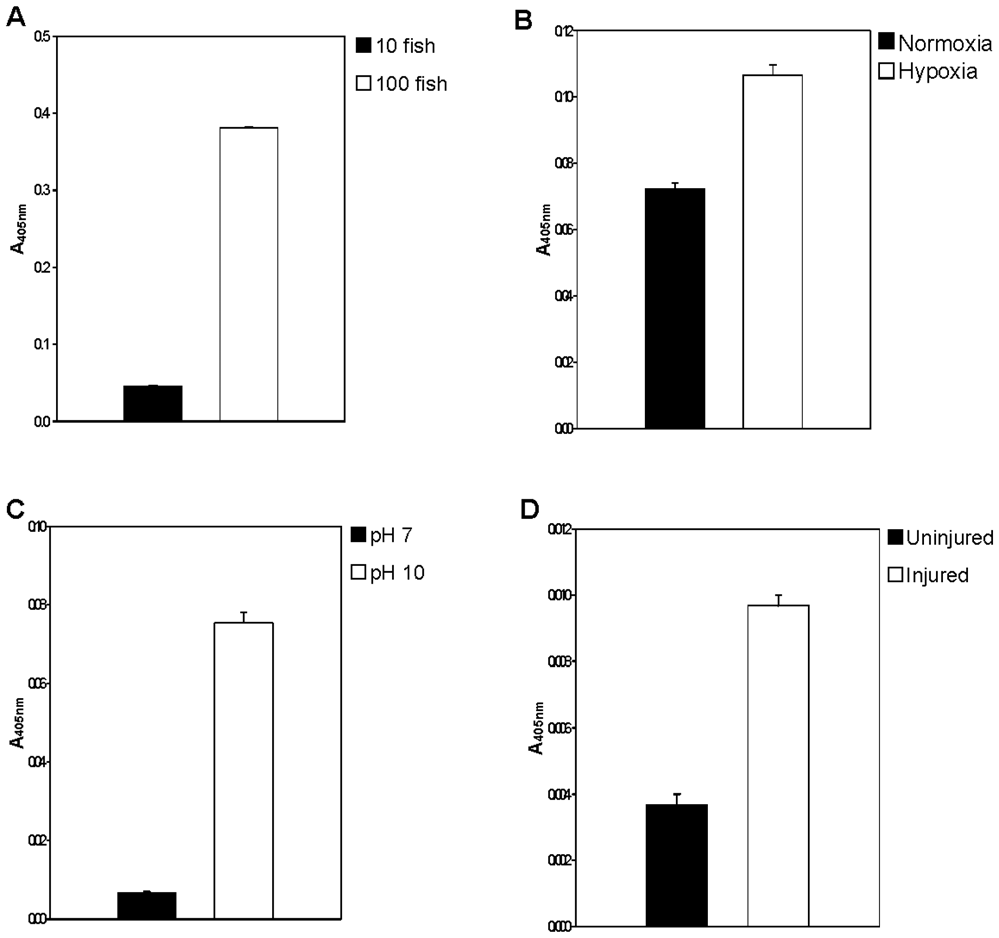
Response of trypsin activity to stresses. (A) To assess the effects of crowding, 10 and 100 zebrafish were kept separately in 400 ml of water for 30 min. The water samples (40 ml) were lyophilized and then resuspended in 1 ml distilled water. Resuspended ZW from these two groups (60 µl and 6 µl respectively) were used in S-2238 cleavage assay. (B) The effects of hypoxia were examined in two fish that were kept in 200 ml water (normoxia) and 200 ml 10% oxygen water (hypoxia) for 30 min. The water samples (40 ml) from each condition was lyophilized and then resuspended in 1 ml distilled water. Resuspended ZW (90 µl) was used in S-2238 cleavage assay. (C) To assess the effects of pH, 50 ml water was adjusted with NH_4_OH to either pH 7 or pH 10 and 5 fish were kept in this water for 30 minutes. The water was immediately neutralized with HCl. From these two samples, 90 µl of water were directly used (without lyophilization) in the S-2238 cleavage assay. (D) To examine the effects of injury on trypsin activity, the head of zebrafish was immersed in 1 ml distilled water in a 1.5 ml microcentrifuge tube. The water samples collected after 10 sec from uninjured and tail-injured fish were lyophilized and resuspended in 200 µl distilled water. Resuspended ZW (90 µl) was used in the S-2238 cleavage assay.

To investigate whether the secretion of trypsins into water was enhanced in response to injury and stress, we employed a variety of stress conditions including crowding, hypoxia, alkaline pH, and injury by needle prick (Figure 4). Under all of these conditions, trypsin release into water was significantly higher than from control fish, suggesting a considerable response to injury or stress. To determine whether the trypsin is released rapidly, ZW was examined ten seconds after the needle prick injury. We found trypsin activity in water in ten seconds. An individual fish on average produced 0.13 ng of trypsin based on a standard curve generated from purified bovine trypsin. No aldolase activity was detected in ZW under these conditions, suggesting that the increase in trypsin activity is not due to cellular damage or death.

### Role of Trypsin in Hemostasis

**Figure 5:**
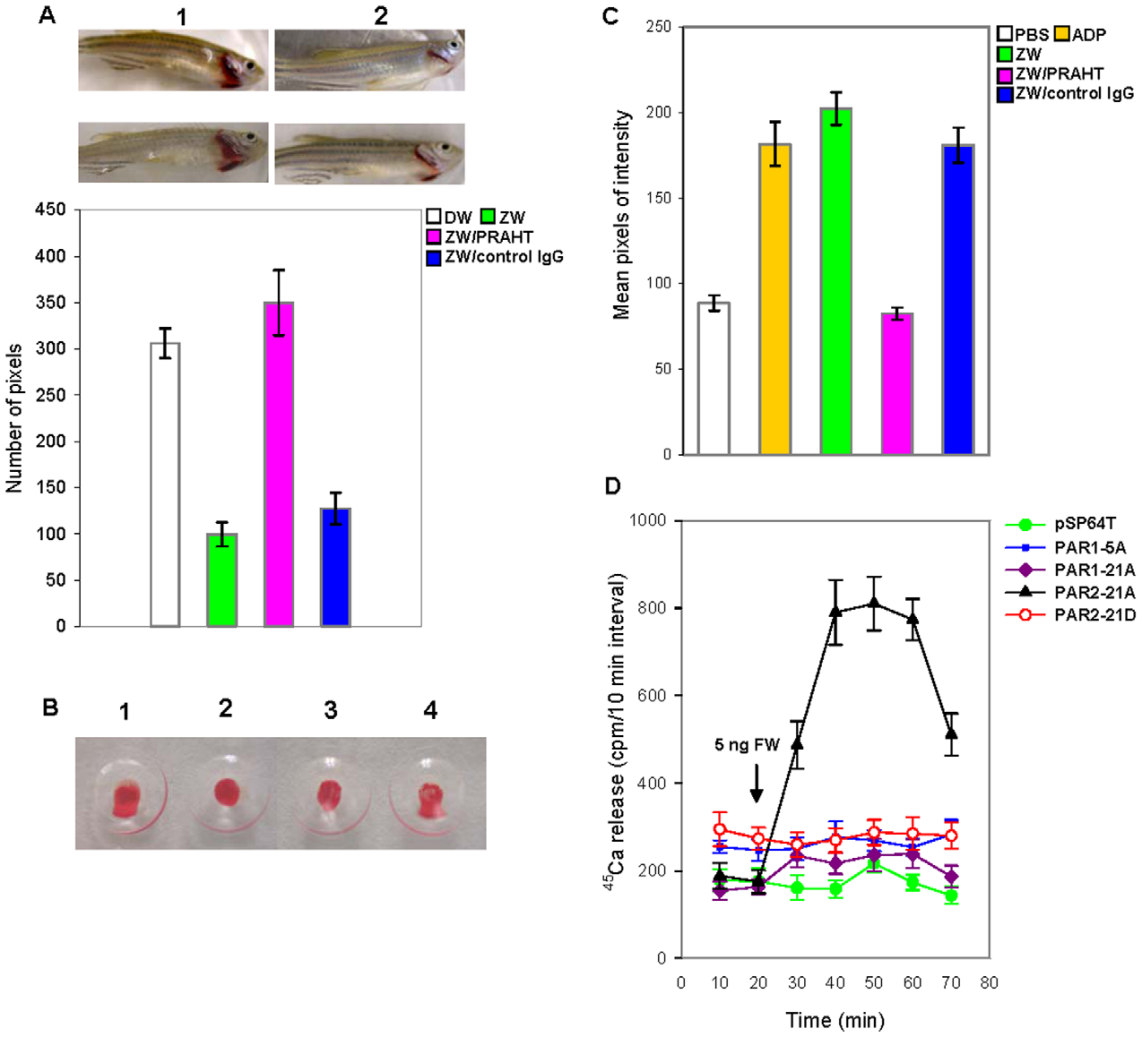
Effects of ZW on gill bleeding, thrombocyte activation, and PAR-induced signaling. (A) The zebrafish were subjected to the gill bleeding assay as described in Materials and Methods with the following conditions: 1, distilled water (DW); 2, 70 ng ZW; 3, 70 ng of ZW with 140 ng of PRAHT; and 4, 70 ng of ZW with 140 ng of control IgG. (B) Plate tilt assay measuring thrombocyte function: 1, PBS; 2, 7 ng ZW; 3, 7 ng ZW with 14 ng control IgG;and 4, 7 ng ZW with 14 ng PRAHT. Note the aggregated blood in 2 and 3 is more firm than the control blood 1 and blood 4. (C) Measurement of the functional activity of ZW on thrombocytes by annexin V binding. 7 ng of ZW, 14 ng of PRAHT and 14 ng of control IgG were used. The intensity of images from FITC-annexin fluorescence (green) was measured as mean pixels using Adobe Photoshop software version 7.0. (T test, n = 12). (D) ZW-induced ^45^Ca^2+^ release from Xenopus oocytes microinjected with PARs mRNA. The data is presented as the mean value and standard error for 12 oocytes for each PAR mRNA.

Enhancement of trypsin production during stress or injury may ultimately be beneficial in protecting the organism. In mammals, stress-induced bleeding occurs in lungs. For example, thoroughbred horses bleed in their lungs during races, and humans have bleeding in their lungs under high altitude hypoxic conditions [13], [14]. The non-coagulation proteases may participate in the breakdown of clotting factors and thus, may regulate extravascular coagulation [15] and participate in fibrinolysis [16]. This proteolytic degradation presents a conundrum because if the fish responds to a bleeding injury by producing more trypsins, hemostatic defense will be compromised and continued bleeding will be favored. To decipher the role of trypsins in hemostasis, we first tested whether the trypsin activity in ZW was inhibited by PRAHT. Indeed, PRAHT inhibited trypsin activity in ZW compared to control IgG (Figure S3). Next, we examined the degree of bleeding following addition of ZW to a zebrafish in water. Addition of ZW to zebrafish significantly reduced levels of bleeding upon alkali-induced injury to gills compared to controls (Figure 5A). Bovine trypsin also reduced bleeding but required higher concentrations of trypsin compared to ZW (Figure S4 and Figure 5A). As a corollary experiment, fish in ZW containing PRAHT exhibited greater bleeding than fish in ZW alone or fish treated with control IgG. This result indicated that trypsin actually controls bleeding. Since higher concentration of bovine trypsin was required to reduce bleeding we suspected that zebrafish trypsins may be more active than bovine trypsin. Therefore, we cloned three zebrafish trypsin full length cDNAs as well as the bovine cDNA into the *E.coli* expression vector pMALc2e and after affinity purification and enterokinase cleavage, trypsin activity was estimated. We found that all three zebrafish trypsins had similar levels of activities and were tenfold more active than bovine trypsin per ng of protein (Figure S5). Next, we determined whether trypsins that are known to activate protease-activated receptors (PARs) on cells could activate blood cells using the thrombocyte aggregation assay. In fact, ZW enhanced cellular aggregation in this assay (Figure 5B), and this activity was inhibited by PRAHT. Annexin V binding assays, which measure thrombocyte activation, revealed that the thrombocytes were indeed activated by ZW, confirming that the observed cellular aggregation was due to activation of thrombocytes (Figure 5C). Furthermore, this activation was inhibited by PRAHT (Figure 5C). In both thrombocyte aggregation assay and annexin V binding assay bovine trypsin activated thrombocytes but not as efficiently as ZW and required higher concentrations than ZW (Figure 5B, 5C and Figure S6, S7). Together, these results established that trypsin present in ZW activates thrombocytes. The level of trypsin produced by the fish is typically sufficient to initiate thrombocyte activation.

### Hemostatic Defense by Trypsin Involves PAR2

**Figure 6:**
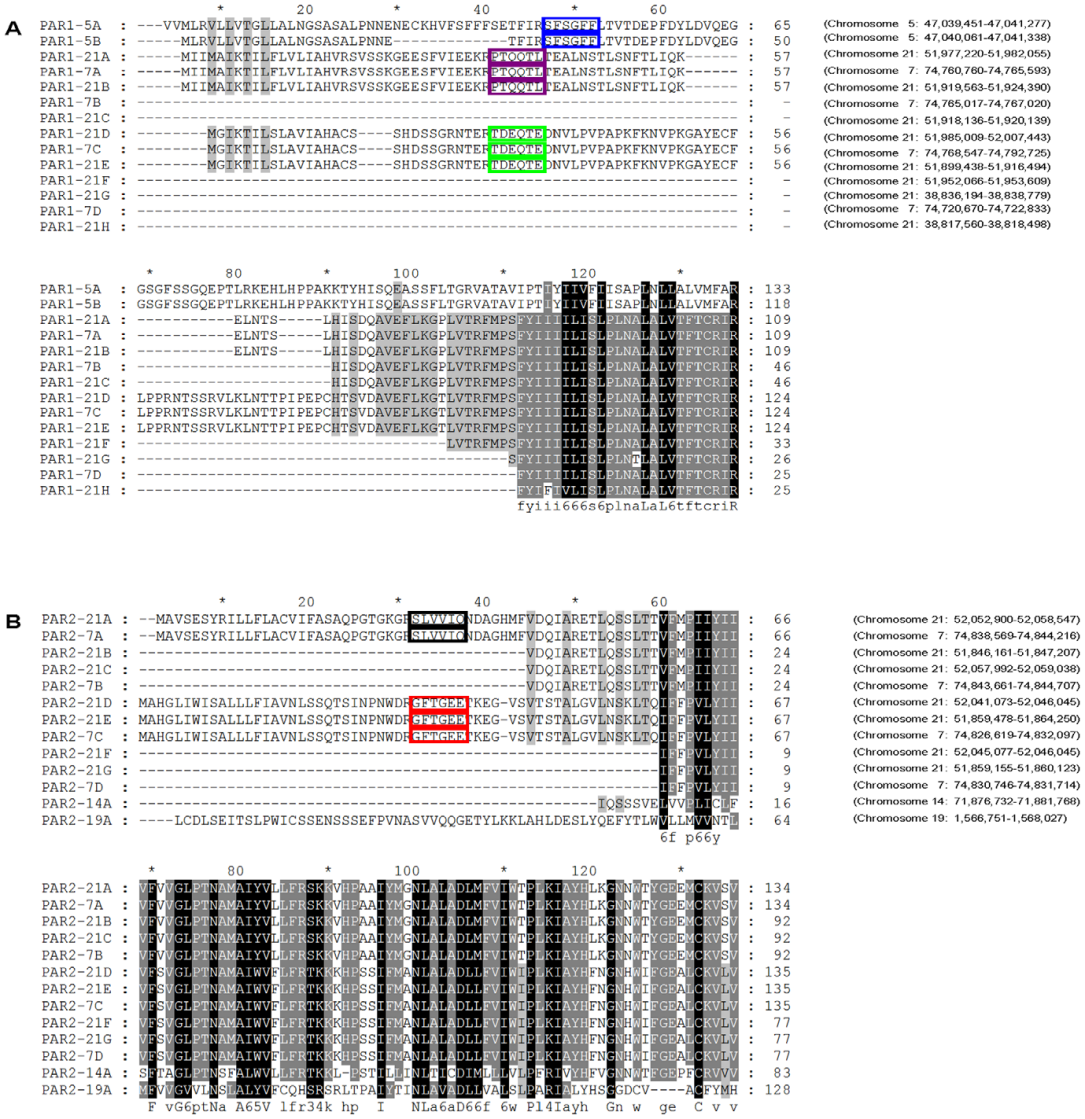
Alignment of amino acids in the PARs. (A and B) The alignment of the amino acids among different groups of PAR1 and PAR2, respectively. The boxed peptides represent the amino acids involved in tethering to activate thrombocytes. The genomic locations are given in parenthesis (Ensembl Danio rerio versions 44.6e, Zv6 and 49.7c, Zv7).

To examine the involvement of PARs in this pathway, we tested whether the Gq inhibitor that blocks the PAR activity would inhibit the action of trypsin [17]. The Gq inhibitor indeed blocked the activity of trypsin in ZW (data not shown). Since four mammalian PARs have been described and PAR2 has been shown to be preferentially activated by trypsin, we investigated whether thrombocyte activation by ZW involves PAR2. Since no information was available for zebrafish PARs, we analyzed the zebrafish genome and identified 27 PAR-like genes. By sequence homology, we classified these PARs into two families: PAR1 and PAR2. These receptors clustered into five groups (three PAR1 groups and two PAR2 groups) based on the tethering peptide sequences. These signature sequences act as PAR agonists and suggest extensive gene duplication (Figure 6). Interestingly, four members (PAR1-21D, PAR1-7C, PAR1-21E, and PAR1-21G) contain more than two exons while most of the other zebrafish PARs contain only two exons similar to those found in humans. Thirteen of these sequences did not contain complete NH2 termini (shown by dotted lines in Figure 6), and therefore, the tethering peptides were not determined for these members. According to RT-PCR analyses, PAR1-5A, PAR1-21A, PAR2-21A, and PAR2-21D were expressed in a pure population of thrombocytes; however, PAR1-21D expression was not detected (Figure S8). The full length cDNAs for PAR1-5A, PAR1-21A, PAR2-21A, and PAR2-21D were cloned into the expression vector pSP64T to generate translatable RNAs, which were then injected individually into Xenopus oocytes [10]. After a few hours, the oocytes were activated by ZW, and the radioactive calcium release of the oocytes was measured as an index of trypsin activation of the expressed PARs. PAR2-21A was activated more than the other PAR members (Figure 5D). In control experiments, human thrombin activated PAR1-5A and PAR1-21A but failed to activate PAR2-21A (Figure S9). These experiments provided evidence that ZW trypsin proteases activate PAR-2 in thrombocytes and play a significant role in primary hemostasis.

**Figure 7:**
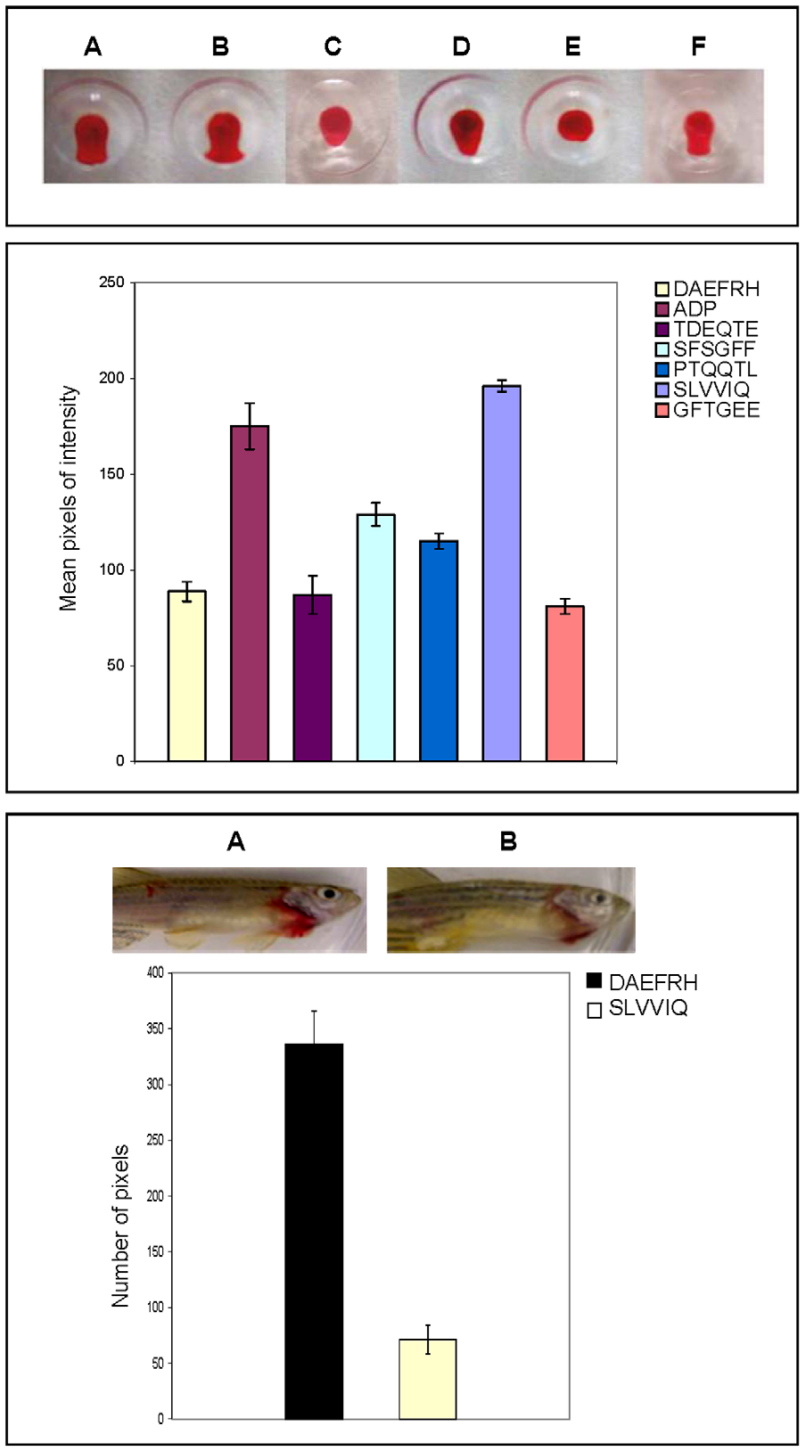
Effect of PAR tethering peptides on thrombocyte function. Top panel: Effect of PAR tethering peptides on thrombocyte aggregation. Plate tilt assay measuring thrombocyte function. (A) DAEFRH (control peptide). (B) TDEQTE (PAR1-21D). (C) SFSGFF (PAR1-5A). (D) PTQQTL (PAR1-21A). (E) SLVVIQ (PAR2-21A). (F) GFTGEE (PAR2-21D). Note the aggregated blood in (C), (D) and (E) is firmer than the control blood (A) and blood (B) and (F). 5 ng of each peptide was used. Middle panel: Measurement of functional activity of PAR tethering peptides on thrombocytes by annexin V binding. 5 ng of each tethering peptide was used. The intensity of images from FITC-annexin fluorescence (green) was measured as mean pixels using Adobe Photoshop software version 7.0 (T test, n = 12). Bottom panel: Effect of SLVVIQ tethering peptides on gill bleeding. The zebrafish were subjected to the gill bleeding assay as described in Materials and Methods with the following conditions: (A) 50 ng of DAEFRH (control peptide) and (B) 50 ng of SLVVIQ (PAR2-21A).

We next synthesized PAR tethering peptides, which mimic protease cleavage and signaling, in order to determine whether the peptides induced signaling. In plate-tilt and annexin V binding assays, the PAR1-21A (PTQQTL) and PAR1-5A (SFSGFF) peptides yielded moderate thrombocyte activation while the PAR2-21A (SLVVIQ) peptides resulted in significant thrombocyte activation (Figure 7). The GFTGEE and TDEQTE peptides failed to induce activity. In addition, SLVVIQ stopped alkali-induced gill bleeding and mimicked trypsin activation (Figure 7). Thus, under stress conditions, fish respond by producing extravascular trypsin and prevent gill bleeding by activating thrombocytes via PAR2 signaling.

### Conclusions

This investigation demonstrates the secretion of trypsins mainly from gills by fishes into the surrounding water in response to injury and activating thrombocytes, providing evidence for their role in hemostasis. Since gills are exposed to the outside environment due to the constant flow of water, and because they are extremely important in vital respiratory function, gills should be protected against injury more than any other organ in the body. In fact, the gill bleeding is a common problem due to toxic ammonia (http://www.algone.com/fish_poisoning.php). In their natural habitat fish with wounded gills are commonly noticed. Since fish gills are highly vascularized and are a point of high blood pressure it is possible that they are evolutionarily selected to produce trypsin to protect them from bleeding before even coagulation cascade is activated. This paper also demonstrates that three trypsins are secreted and they are more active than bovine tryspin. This finding raises important questions such as what is the mechanism of trypsin secretion. Is trypsin stored as trypsinogen and then cleaved by another enzyme when it is secreted or is trypsin stored to similar to mast cell tryptases and then released when under injury [18]. Thus, we predict that this work opens new doors for future investigations of these studies.

Also, we speculate the trypsin may not be exclusively protecting the gills from bleeding but may also protect the fish from bleeding from other body injuries. This protection theoretically stems from the fact that fish in locomotion produce von Karman Vortex Street similar to what is produced by torpedoes when they move [19]. Earlier workers using zebrafish adults and larvae and 20–40 µm polystyrene spheres in particle image velocimetry, established the flow fields and vortices and their diameters [20]. Based on this study, any molecule should follow the physical principles of hydrodynamic motion. As a result of the von Karman Vortices, even a few molecules of trypsin produced by the gills will quickly be in close contact with the body. We estimated about 0.13 ng trypsin is produced during a ten second period from an injured fish. Taking the vortex core radius as 0.5 cm and the maximum height of the vortex cylinder (body width of zebrafish) as 0.5 cm, the core cylinder volume will be about 0.4 ml volume in a given vortex. Therefore, the concentration of trypsin in the core cylinder would be 0.325 ng/ml, which is approximately 1/10^th^ of the circulating VIIa concentration in mammals that is sufficient to initiate blood clotting when the stream of blood is leaking from the vessel during injury. In reality, these vortex cylinder heights are not as big as the body width and are usually small. Similarly, the amounts of trypsin released may vary depending upon the number of vortices generated per second. Despite these variations, since fish trypsins are far more active than mammalian trypsins, the trypsin following the von Karman Streets must be effective in activating thrombocytes. During thrombosis, thrombus forms even though the blood is flowing and this blood flow does not wash away the thrombus (unless the clot is weak). Similarly in gills where there is laminar flow of water, trypsin is very effective and subsequently when it exits the gills, it assumes the complex hydrodynamic flow where the moving vortex of trypsin water will activate the thrombocytes. Thus, binding of trypsin to the PAR-2, in order to enhance thrombocyte signaling is not difficult to conceive in moving vortex and particularly since initial thrombocytes are anchored to the subendothelial surface (via collagen and von Willebrand factor). Along the body musculature, obviously, the vortex helps prevent trypsin to be diluted away by the water, particularly since fish generates suction pressures due to the vorteces along the fish body. In both gills and on body musculature, in addition to thrombocyte activation by trypsin that initiates primary hemostasis, clotting cascade triggered by VIIa-tissue factor interactions will generate thrombin subsequently and take over the primary hemostasis to complete the plugging of the injured area by generation of fibrin (secondary hemostasis). It should be noted that the body and gills are protected by mucus and, thus, they are protected from trypsin action similar to the protection of digestive tract by mucus cells.

In addition to trypsin we also found antitrypsin in our MALDI-TOF analysis. This finding would make sense because if excess of trypsin is produced it may be damaging the exposed vessels during injury. Therefore, production of antitrypsin seems to control the trypsin activity. However, observed trypsin activity in the complexes is puzzling. One explanation is that during incubation in gel, trypsin could dissociate from the complex could give activity. Such release of complexes has been noted earlier. Interestingly, we noted that the 55–75 kDa band likely appears as a smear. This is because there are multiple trypsins and serpins along with their possible degradation products and thus different size complexes are possible. Also, the complexes seem to be covalently linked because these proteins have been run on denaturing polyacrylamide gels. This is consistent with the finding that covalent complexes do form between serpins and serine proteases [21].

The PARs are susceptible for proteolytic cleavage which sets the signal transduction in various types of cells including platelets. In humans there are four PARs and two of them are expressed in human platelets, PAR1 and PAR4. In mouse platelets PAR3 and PAR4 seem to play a role in thrombin signaling. This paper documents that there are 27 PARs which appear to be products derived from tandemly duplicated genes. We could only classify them based on the tethering peptides into two families, PAR1 and PAR2. Thus, PAR3 and PAR4 seem to have evolved later in evolution. Interestingly PAR1 in fish are activated by thrombin while PAR2 is activated by trypsin. Thus, this investigation clearly documents distinct thrombin and trypsin receptors.

In summary, we report here trypsins are released by fish into water in response to injury and stress conditions in order to protect the fish from bleeding. This finding provides evidence for the role of trypsins in primary hemostasis of early vertebrates.

## Supporting Information

Figure S1 Kinetic analysis of the S-2238 cleaving activity in zebrafish water. (A) Lineweaver Burk plot with V against l/[S]. (B) Effect of pH on enzymatic activity. MES (50 mM 2-morpholinoethanesulphonic acid, pH 5–7) and BTP (50 mM bis-Tris-propane, pH 7–9.5) were used to replace Tris-HCl buffer to study the effect of pH. (C–G) Effect of temperature, SDS, urea, CaCl2, and EDTA on enzymatic activity, respectively. (B–G) Each sample was kept for 30 min prior to mixing with substrate under the above specified conditions. Standard error bars (±0.07) were removed to improve clarity of each graph.
(0.33 MB TIF)

Figure S2 RT-PCR of trypsins in gill, mouth, and nose from zebrafish. Tissues from each organ were obtained using microscissors. RNA from tissues was isolated using Absolutely RNA prep kit from Stratagene, Inc. Trypsin primers were designed using MALDI-TOF data and NCBI database (Forward 5′-TCATGCTGATCAAGCTGA-3′ Reverse 5′-ATCCAGCGCAGAACATG-3′).
(0.08 MB TIF)

Figure S3 Effect of PRAHT on S-2238 cleavage activity of ZW. S-2238 cleavage activity assay was performed using 70 ng of ZW in a final assay volume of 200 µL containing 500 µM S-2238 in 50 mM Tris-HCl pH 7.2. Control IgG (140 ng) and polyclonal rabbit anti-human trypsin (PRAHT) antibody (140 ng) were used in this assay. Inclusion of fourfold excess of PRAHT completely abolished trypsin activity (data not shown).
(0.00 MB TIF)

Figure S4 Effect of trypsin on gill bleeding. The zebrafish were subjected to the gill bleeding assay as described in the Methods with the following conditions: (A) Distilled water (DW), (B) 200 ng bovine trypsin, (C) 100 ng of bovine trypsin with 200 ng of PRAHT, and (D) 200 ng of bovine trypsin with 400 ng of control IgG.
(0.80 MB TIF)

Figure S5 The purified trypsins were separated on a 5–20% Tris-glycine SDS-polyacrylamide gradient gel. The values in the bottom panel represent the S-2238 cleaving activity of 1 ng of each sample at A_405nm_ and the mean of three independent determinations ± the standard error.
(0.30 MB TIF)

Figure S6 Effect of bovine trypsin on thrombocyte aggregation. Plate tilt assay measuring thrombocyte function. (A) PBS. (B) 20 ng of bovine trypsin. (C) 20 ng of bovine trypsin with 40 ng PRAHT. (D) 20 ng of bovine trypsin with 40 ng control IgG. Note the aggregated blood in (B) and (D) is more firm than the control blood (A) and blood (C).
(0.27 MB TIF)

Figure S7 Measurement of functional activity of bovine trypsin on thrombocytes by annexin V binding. 20 ng of bovine trypsin, 40 ng of PRAHT and 40 ng of control IgG were used. The intensity of images from FITC-annexin fluorescence (green) was measured as mean pixels using Adobe Photoshop software version 7.0 (T test, n = 12).
(0.36 MB TIF)

Figure S8 RT-PCR for detection of the PAR receptors on thrombocytes. Thrombocytes were collected using nanoject II. RNA was isolated from the thrombocytes (n = 455) using Absolutely RNA prep kit from stratagene, Inc. Primers: GpIIb (Forward 5′- CAGCTGGACAGAATGAAGCA-3′ Reverse 5′- GGGAGTCAGCCAAGCTGTAG-3′), PAR1-21D (Forward 5′-ACCTTGTTGTATCACTGTGT-3′ Reverse 5′-TTTCTGTAATGAGATGAACC-3′), PAR1-5A (Forward 5′-TTACCTGTACTTCTTTCCAA-3′ Reverse 5′-AAAACTGCAAAAACTGTTAC-3′), PAR1-21A (Forward 5′-AACAATCTTGTTTTTAGTGC-3′ Reverse 5′-ATGATGATGATGTAGAAGGA-3′), PAR2-21A (Forward 5′-GAGATGTGCAAAGTATCAGT-3′ Reverse 5′-ACGGTTTGATTGTAGAGATA-3′), PAR2-21D (Forward 5′-CTCTGTATCTTTACGACCAG-3′ Reverse 5′-CACGTTTGACACTATACACA-3′).
(0.35 MB TIF)

Figure S9 Effect of thrombin on PAR-induced signaling. Thrombin-induced ^45^Ca^2+^ release from Xenopus oocytes microinjected with PARs mRNAs. The data is presented as the mean value and standard error for 12 oocytes for each PAR mRNA.
(0.32 MB TIF)

Table S1 Peptide sequences identified from MALDI-TOF analysis of the 25 kDa band as described in Materials and Methods.
(0.04 MB DOC)

Table S2 Peptide sequences identified from MALDI-TOF analysis of the 55–75 kDa band as described in Materials and Methods.
(0.15 MB DOC)
