# Supplementary figures and images for: Evolution of Primary Hemostasis in Early Vertebrates
Source: PLoS One. 2009 Dec 23;4(12):e8403. doi: 10.1371/journal.pone.0008403 (PMC2793433; doi:10.1371/journal.pone.0008403)

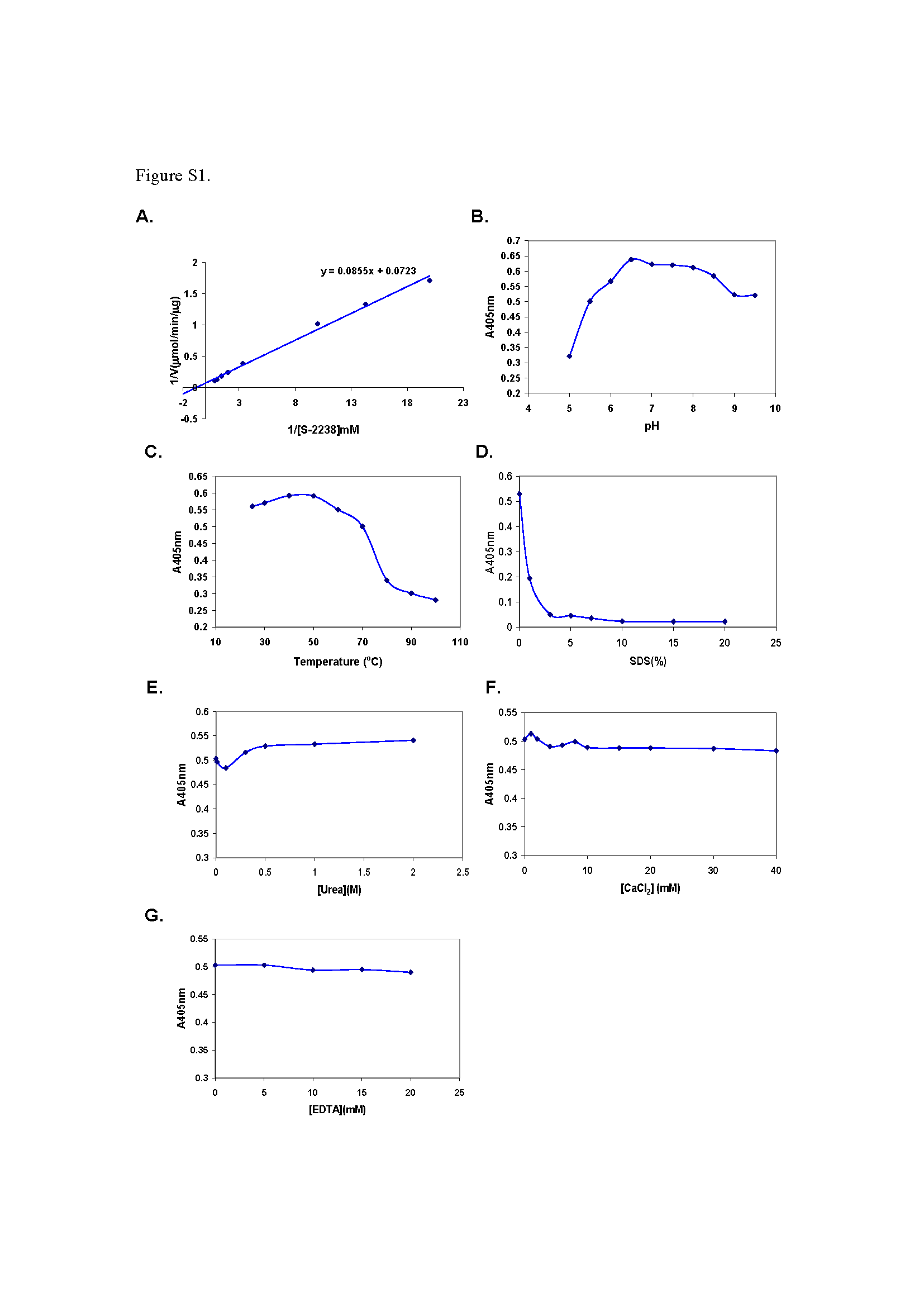

Supplement: Figure S1 — Kinetic analysis of the S-2238 cleaving activity in zebrafish water. (A) Lineweaver Burk plot with V against l/[S]. (B) Effect of pH on enzymatic activity. MES (50 mM 2-morpholinoethanesulphonic acid, pH 5–7) and BTP (50 mM bis-Tris-propane, pH 7–9.5) were used to replace Tris-HCl buffer to study the effect of pH. (C–G) Effect of temperature, SDS, urea, CaCl2, and EDTA on enzymatic activity, respectively. (B–G) Each sample was kept for 30 min prior to mixing with substrate under the above specified conditions. Standard error bars (±0.07) were removed to improve clarity of each graph. (0.33 MB TIF) [file pone.0008403.s001.tif]

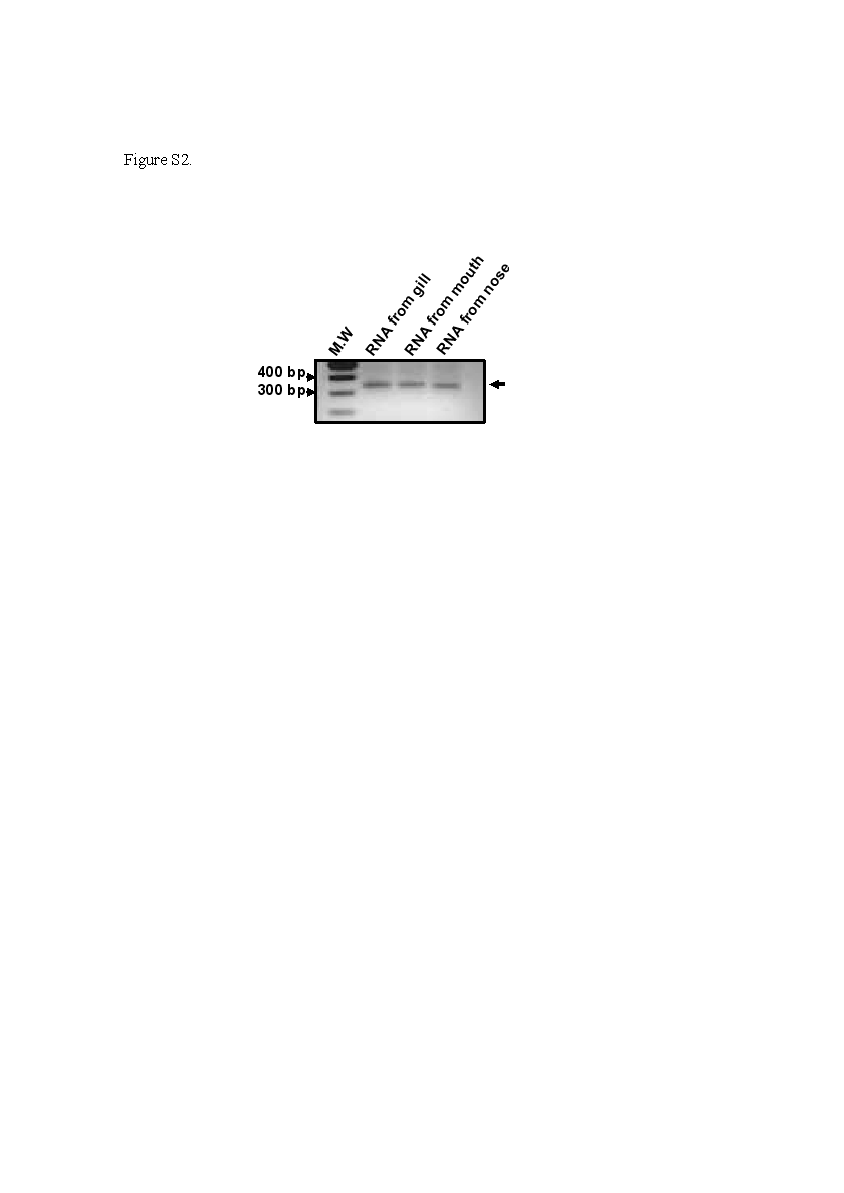

Supplement: Figure S2 — RT-PCR of trypsins in gill, mouth, and nose from zebrafish. Tissues from each organ were obtained using microscissors. RNA from tissues was isolated using Absolutely RNA prep kit from Stratagene, Inc. Trypsin primers were designed using MALDI-TOF data and NCBI database (Forward 5′-TCATGCTGATCAAGCTGA-3′ Reverse 5′-ATCCAGCGCAGAACATG-3′). (0.08 MB TIF) [file pone.0008403.s002.tif]

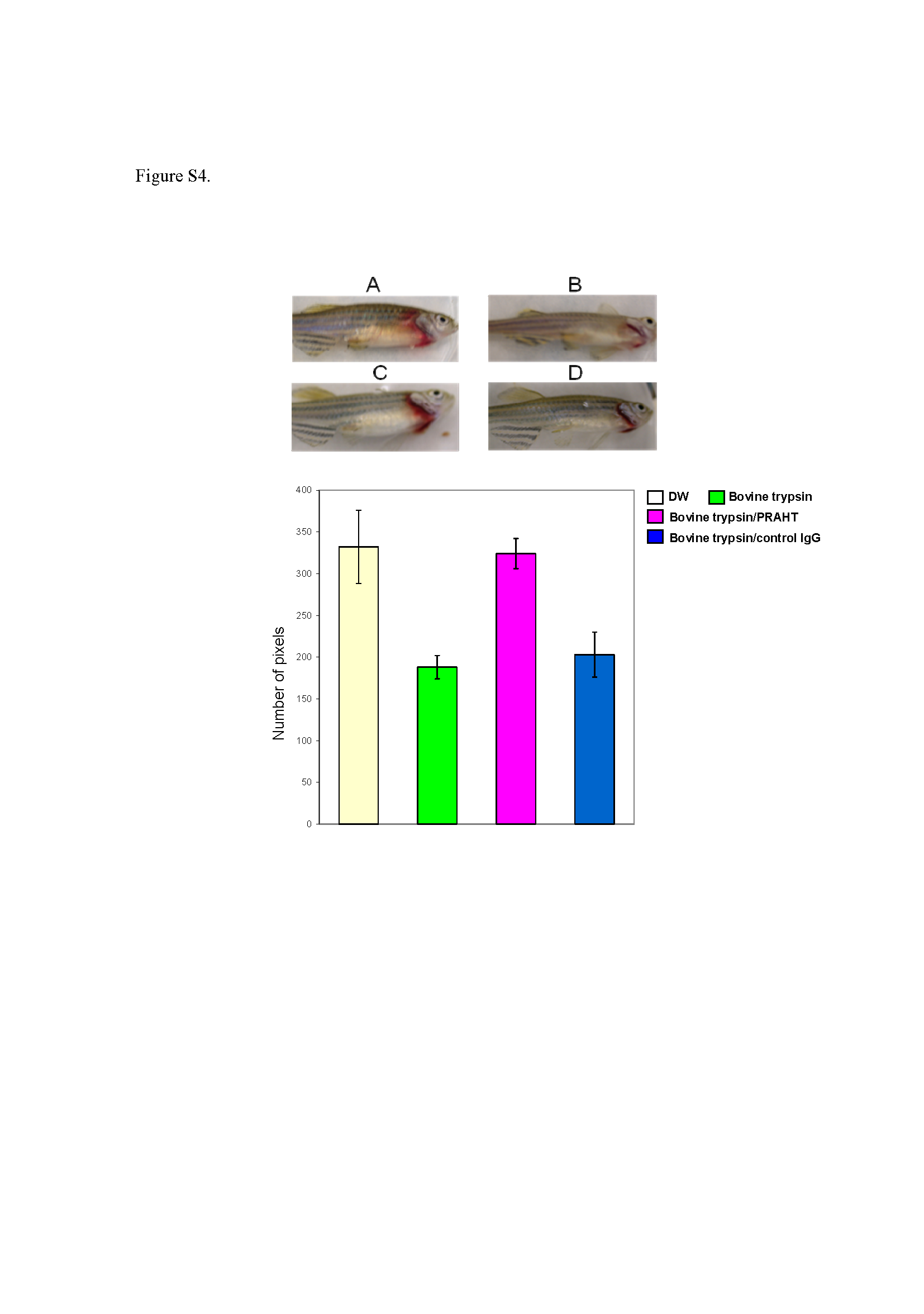

Supplement: Figure S4 — Effect of trypsin on gill bleeding. The zebrafish were subjected to the gill bleeding assay as described in the Methods with the following conditions: (A) Distilled water (DW), (B) 200 ng bovine trypsin, (C) 100 ng of bovine trypsin with 200 ng of PRAHT, and (D) 200 ng of bovine trypsin with 400 ng of control IgG. (0.80 MB TIF) [file pone.0008403.s004.tif]

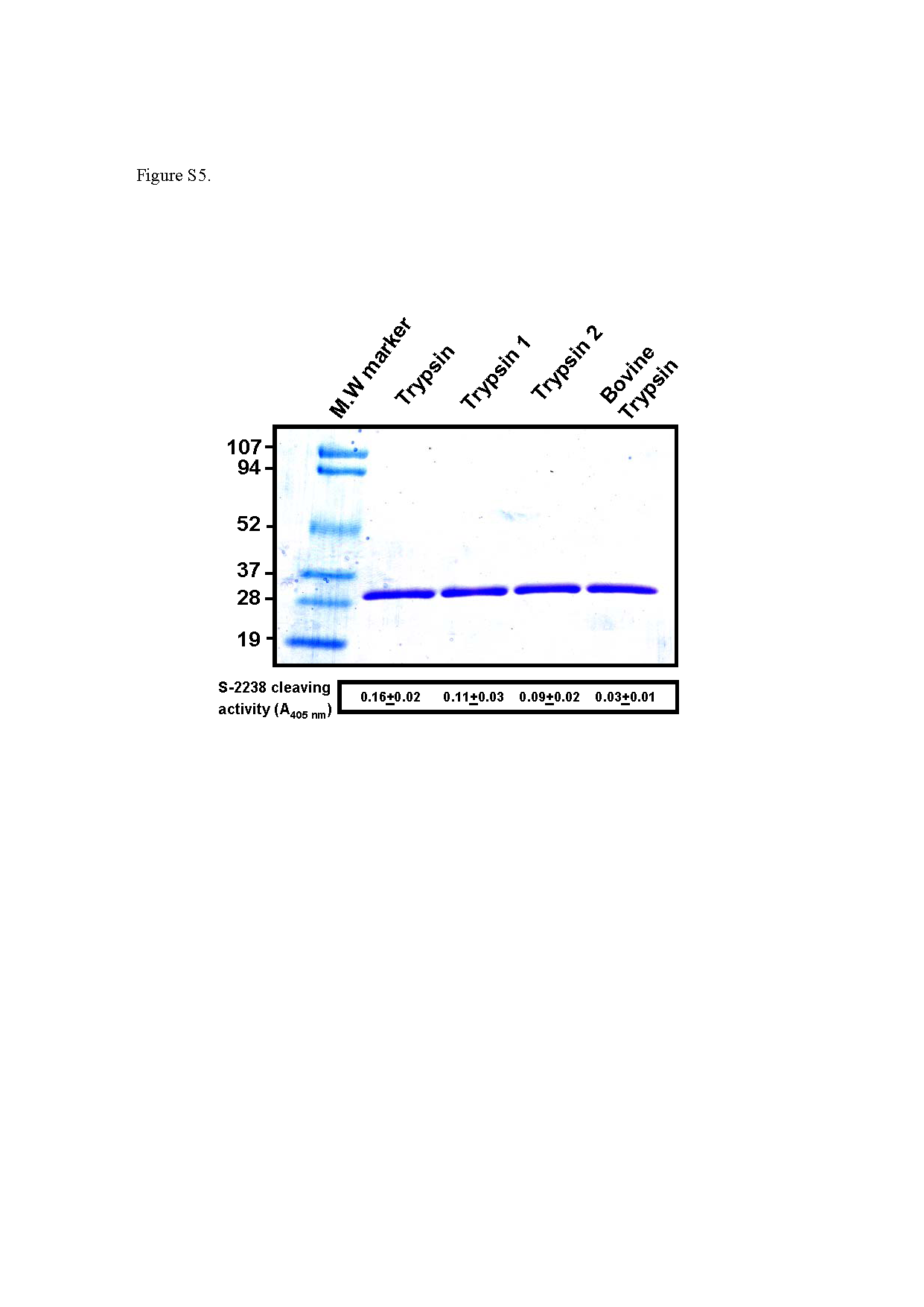

Supplement: Figure S5 — The purified trypsins were separated on a 5–20% Tris-glycine SDS-polyacrylamide gradient gel. The values in the bottom panel represent the S-2238 cleaving activity of 1 ng of each sample at A405nm and the mean of three independent determinations ± the standard error. (0.30 MB TIF) [file pone.0008403.s005.tif]

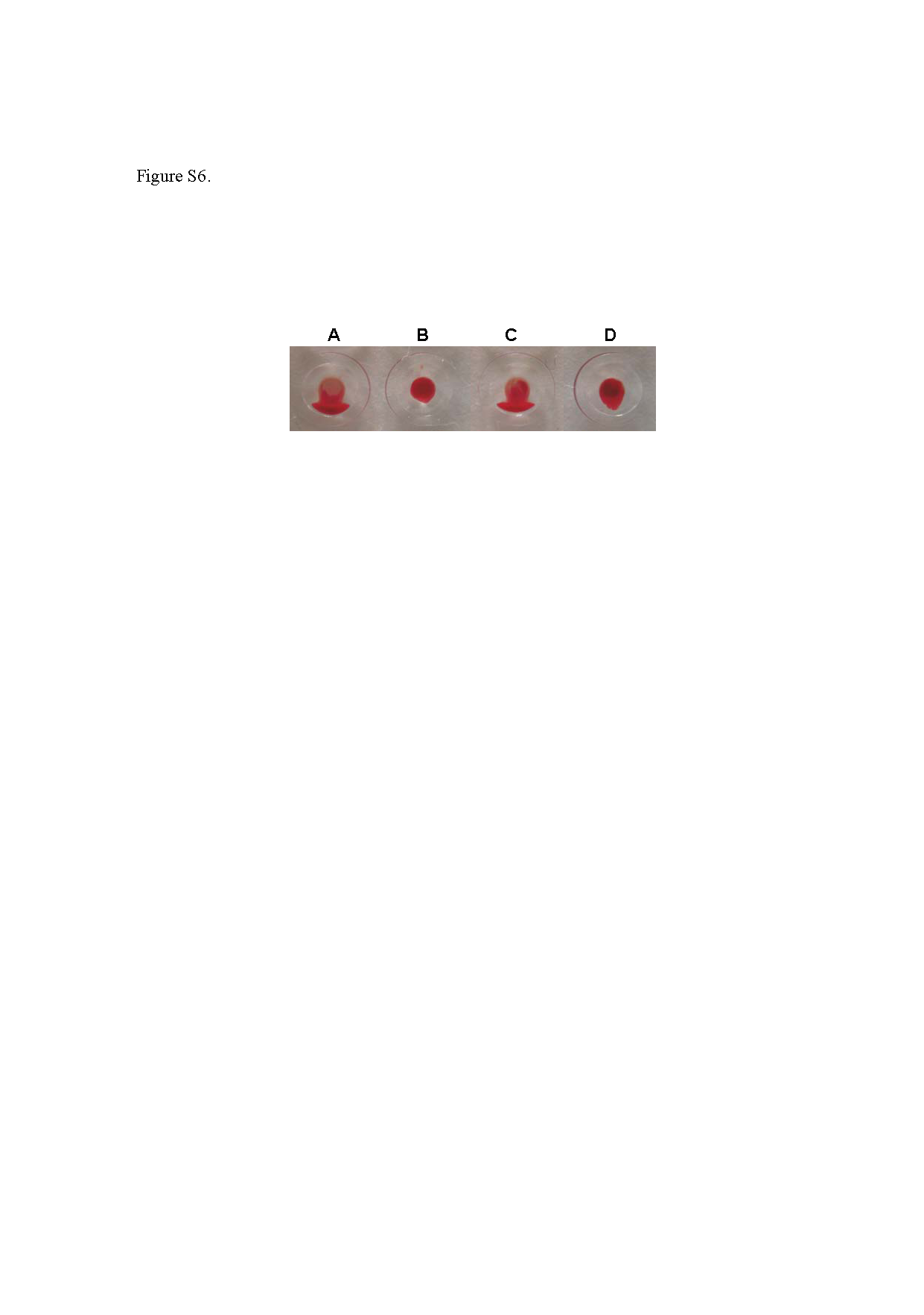

Supplement: Figure S6 — Effect of bovine trypsin on thrombocyte aggregation. Plate tilt assay measuring thrombocyte function. (A) PBS. (B) 20 ng of bovine trypsin. (C) 20 ng of bovine trypsin with 40 ng PRAHT. (D) 20 ng of bovine trypsin with 40 ng control IgG. Note the aggregated blood in (B) and (D) is more firm than the control blood (A) and blood (C). (0.27 MB TIF) [file pone.0008403.s006.tif]

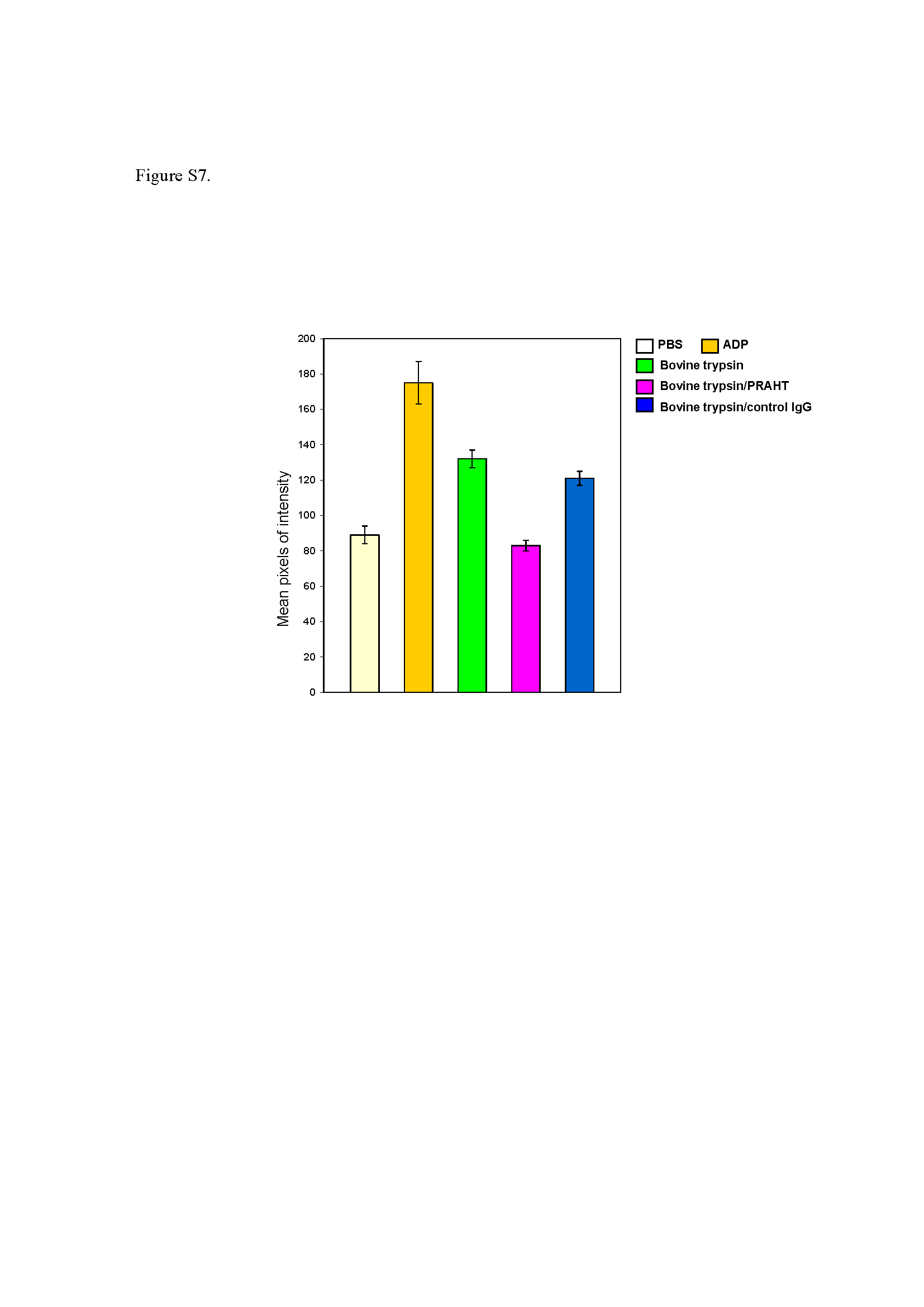

Supplement: Figure S7 — Measurement of functional activity of bovine trypsin on thrombocytes by annexin V binding. 20 ng of bovine trypsin, 40 ng of PRAHT and 40 ng of control IgG were used. The intensity of images from FITC-annexin fluorescence (green) was measured as mean pixels using Adobe Photoshop software version 7.0 (T test, n = 12). (0.36 MB TIF) [file pone.0008403.s007.tif]

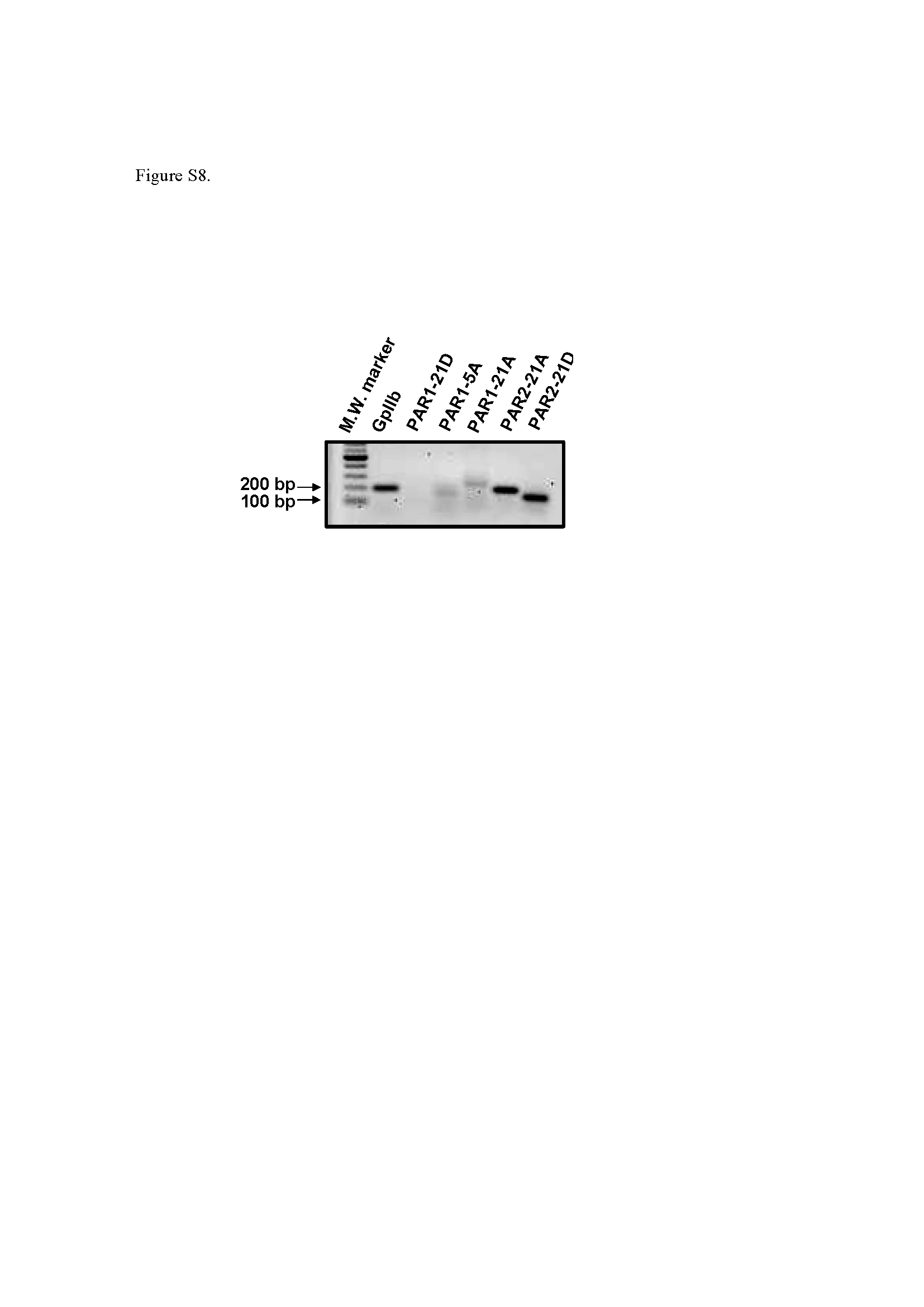

Supplement: Figure S8 — RT-PCR for detection of the PAR receptors on thrombocytes. Thrombocytes were collected using nanoject II. RNA was isolated from the thrombocytes (n = 455) using Absolutely RNA prep kit from stratagene, Inc. Primers: GpIIb (Forward 5′- CAGCTGGACAGAATGAAGCA-3′ Reverse 5′- GGGAGTCAGCCAAGCTGTAG-3′), PAR1-21D (Forward 5′-ACCTTGTTGTATCACTGTGT-3′ Reverse 5′-TTTCTGTAATGAGATGAACC-3′), PAR1-5A (Forward 5′-TTACCTGTACTTCTTTCCAA-3′ Reverse 5′-AAAACTGCAAAAACTGTTAC-3′), PAR1-21A (Forward 5′-AACAATCTTGTTTTTAGTGC-3′ Reverse 5′-ATGATGATGATGTAGAAGGA-3′), PAR2-21A (Forward 5′-GAGATGTGCAAAGTATCAGT-3′ Reverse 5′-ACGGTTTGATTGTAGAGATA-3′), PAR2-21D (Forward 5′-CTCTGTATCTTTACGACCAG-3′ Reverse 5′-CACGTTTGACACTATACACA-3′). (0.35 MB TIF) [file pone.0008403.s008.tif]

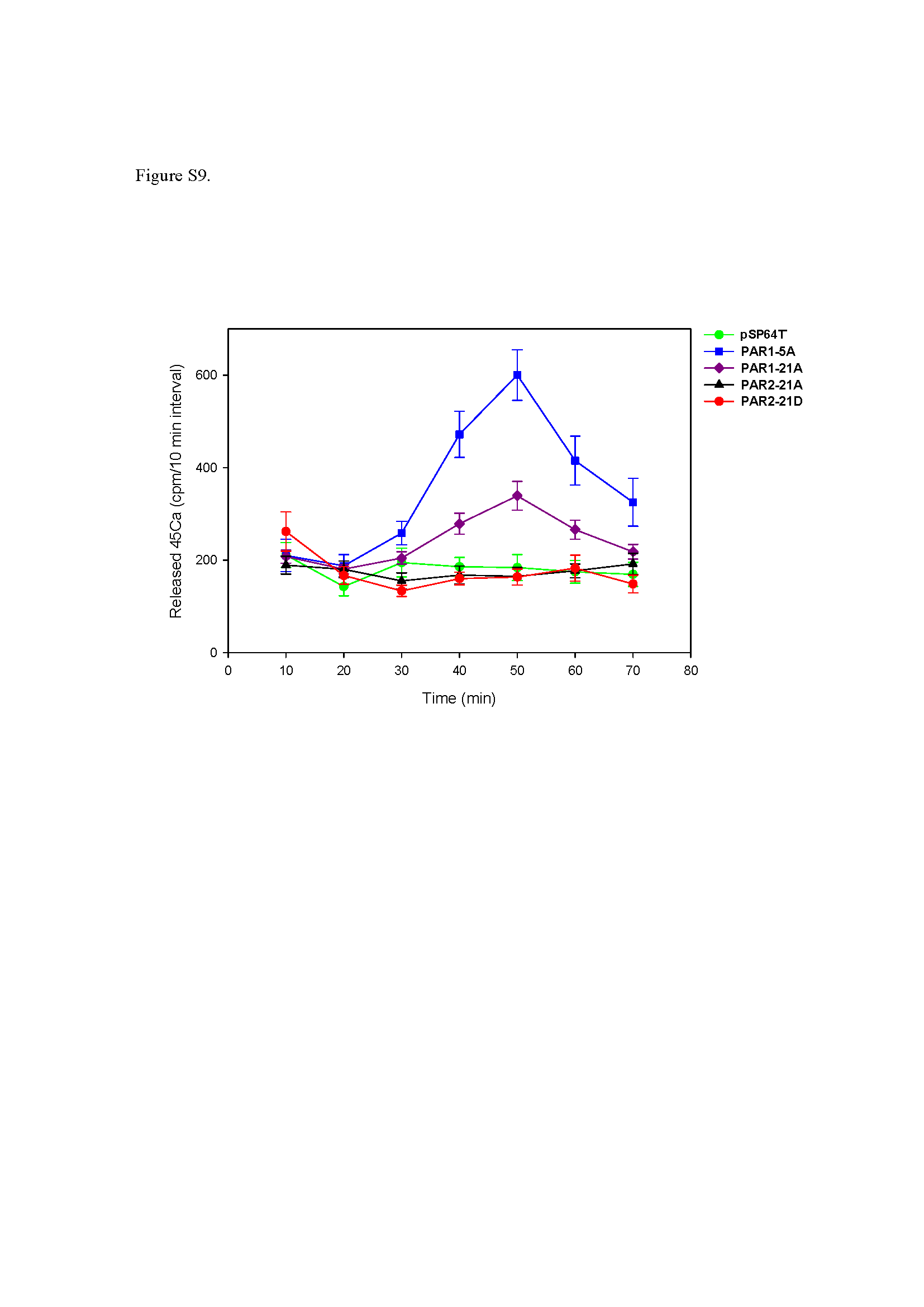

Supplement: Figure S9 — Effect of thrombin on PAR-induced signaling. Thrombin-induced 45Ca2+ release from Xenopus oocytes microinjected with PARs mRNAs. The data is presented as the mean value and standard error for 12 oocytes for each PAR mRNA. (0.32 MB TIF) [file pone.0008403.s009.tif]
