# Supplementary material for: Evolution of Primary Hemostasis in Early Vertebrates
Source: PLoS One. 2009 Dec 23;4(12):e8403. doi: 10.1371/journal.pone.0008403 (PMC2793433; doi:10.1371/journal.pone.0008403)
Supplement: Table S1 — Peptide sequences identified from MALDI-TOF analysis of the 25 kDa band as described in Materials and Methods. (0.04 MB DOC) [file pone.0008403.s010.doc]

Table S1.

| **Protein Name** | **MW (kDa)** | **Peptide Sequence** |
| --- | --- | --- |
| Trypsin | 25.8 | | HPSYNSNTLDNDVMLIK | | --- | | LGEHNIDVTEGTEQFINSEK | | LSSSAQINSYVK | | TVSLPSSCASSGTSCLISGWGNMSASGS | | NYPSR | |
| Trypsin 1 | 26.4 | | DCNNSYPGMVTDTMFCAGYLEGGK | | --- | | LGEHNIVINEGTEQFITSEK | | LQCLEIPILSDR | | LSKPATLNK | | NHPGVYGK | | NPNYDSWDLDSDIMLIK | | VEVRLGEHNIVINEGTEQFITSEK | | VSGWGNTMSSTADSNK | | YVQPVALPNGCAADGTMCR | |
| Trypsin 2 | 27.1 | | DACQGDSGGPVICNGELR | | --- | | LTVHLGEHNVAVEEGTEQR | | YNDYTLDNDFMLIK | | YTDWVASTIANN | |
| Superoxide dismutase 1 | 15.9 | | GTGEVTGTVYFNQEGEK | | --- | | HVGDLGNVTADASGVAK | | IEIEDAMLTLSGQHSIIGR | | VTGEITGLTPGK | |
| Zinc metalloprotease | 29.1 | | DNHIQVIWENILDDMK | | --- | | DYISIESR | | ELEIIQR | | NGLPTMIPIPNNNAALGTSTEMSQNDIIR | | QGYAQTVSLAR | |
