# Supplementary material for: Evolution of Primary Hemostasis in Early Vertebrates
Source: PLoS One. 2009 Dec 23;4(12):e8403. doi: 10.1371/journal.pone.0008403 (PMC2793433; doi:10.1371/journal.pone.0008403)
Supplement: Table S2 — Peptide sequences identified from MALDI-TOF analysis of the 55–75 kDa band as described in Materials and Methods. (0.15 MB DOC) [file pone.0008403.s011.doc]

Table S2.

| **Protein Name** | | **MW (kDa)** | | **Peptide Sequence** | |
| --- | --- | --- | --- | --- | --- |
| Transferrin | | 73.5 | | AGSGFNINELK | | --- | | AVSEFFSSSCVPGVSKPK | | DLIFSDSATELMLLPK | | EADFLAVDGGQVYIAGK | | EEYYEAMQAFK | | EEYYEAMQAFKDGNPSAPTSQTK | | GSGLTWNNLEGK | | GSVDDFLTKK | | HTVVGDYTDGK | | ITWEGPNEMPVER | | KEADFLAVDGGQVYIAGK | | KGSGLTWNNLEGK | | KYIDMIER | | LIATNK | | NLLFTDGTK | | NNDLFTSK | | QIPASDLFSSQAFGGK | | RTDSLLYLK | | SAVVSFLSDIQSK | | TDSLLYLKEEYYEAMQAFK | | TDTDLQYVYDVLK | | TSQNVPDLVK | | | |
| Transferrin-a | | 56.8 | | AVSEFFSSSCVPGVSKPK | | --- | | CKPSAEEQYYGYDGAFR | | DLIFSDSATELMLLPK | | EADFLAVDGGQVYIAGK | | EEYYEAMQAFK | | EEYYEAMQAFKDGNPSAPTSQTK | | GSGLTWNNLEGK | | GSVDDFLTKK | | HTVVGDYTDGK | | KEADFLAVDGGQVYIAGK | | KGSGLTWNNLEGK | | KYIDMIER | | NLLFTDGTK | | NNDLFTSK | | QIPASDLFSSQAFGGK | | RTDSLLYLK | | SAVVSFLSDIQSK | | TDSLLYLKEEYYEAMQAFK | | TDTDLQYVYDVLK | | TSQNVPDLVK | | | |
| Amylase, α-2A 1 | | 56.9 | | ALVFVDNHDNQR | | --- | | CNTGSGNIENYQDINQVR | | DFPTVPYSNLDFNDGK | | EVNVGGDGK | | GHGAGGASIVTFWDAR | | HMWPGDLSAVYGR | | ISNQEEDPFIAIHADSK | | ISNQEEDPFIAIHADSKL | | LHNLNTR | | LIDMGVAGFR | | LVGLLDLALEK | | LVGLLDLALEKDYVR | | NIVNGQDQNDWIGPPSNGDGSTKPVPINPDST- CGDNWVCEHR | | NVVNGQPFSNWWDNGSNQIAFSR | | NWGEGWGFMPSDK | | SGNENELKDMITR | | TAIVHLFEWR  YLGPNGFGGVQISPPSESIVVTNPWHPWWQR | | | |
| Antitrypsin | | 47.8 | | ADFSGMTEEVK | | --- | | DAQHYYNSEAFGVDFSKPEIAAAEINK | | DLDADTVMMLINYMYFR | | LDDILMDMGMTDAFDYK | | NIFFSPVGISMALSLLAVGAK | | VDQDTTVQVDMMK | | VLHQAVMSVDEK | | | |
| Amylase, α-2A 2 | | 56.8 | | ALVFVDNHDNQR | | --- | | CNTGSGNIENYQDINQVR | | EVNVGGDGK | | GHGAGGASIVTFWDAR | | HMWPGDLSAVYGR | | ISNQEEDPFMAIHADSK | | ISNQEEDPFMAIHADSKL | | LHNLNTR | | LIDMGVAGFR | | LVGLLDLALEK | | LVGLLDLALEKDYVR | | NIVNGQDQNDWIGPPSNGDGSTKPVPINPDST- CGDNWVCEHR | | NVVNGQPFSNWWDNGSNQIAFSR | | SGNENELKDMITR | | | |
| Serpin a1 | | 48.3 | | ADFSGMTEEVK | | --- | | DAQHYYNSEAFGVDFSKPEIAAAEINK | | DLDADTVMMLINYMYFR | | DMGMTDAFNDKADFSGMTEEVK | | NIFFSPVGISMALSLLAVGAK | | VDQDTTVQVDMMK | | | |
| Chitinase | | 52.6 | | ATGNPQLMLTAAVSAGK | | --- | | ELVAAYEAESK | | FTGHNSPLYQGSKDEGDLIYFNTDYAMR | | GTIDDGYEIAEIAK | | LTSSDTSVGAPASGPASAGTYTR | | NELVTYEWNDETLYK | | NSEWVGFDTK | | THGFDGLDLDWEYPGAR | | | |
| β-hexosaminidase 1 | | 61.5 | | FMNQQGFGTDYSK | | --- | | GLETFSQLVYEDDYGVR | | LKDDTVVEVWK | | LWSDASVTDVGNAYTR | | TDESYSLSVDETSAVLK | | VEPLDFTGTDAQK | | VVAEFDTPGHTQSWGNGIK | | YYKVEPLDFTGTDAQK | | | |
| β-hexosaminidase 2 | | 61.5 | | FMNQQGFGTDYSK | | --- | | GLETFSQLVYEDDYGVR | | LWSDASVTDVGNAYTR | | TDESYSLSVDETSAVLK | | VSYGQDWVDIYK | | VVAEFDTPGHTQSWGNGIK | | | |
| Amylase α-2A 3 | | 57.5 | | ALVFVDNHDNQR | | --- | | LEDLLDLALEK | | NVVNGQPLFNWWDNGNSQIAFSR | | NWGEGWGFMPSDK | | SASGQIESYNDIYQVR | | SGTEAELKDMITR | | VAEYLNK | | YLAPNGYGGVQISPPSEHVK | | | |
| Trypsin 1 | | 26.4 | | LGEHNIVINEGTEQFITSEK | | --- | | NPNYDSWDLDSDIMLIK | | VSGWGNTMSSTADSNK | | | |
| Cpb1 protein | | 46.4 | | MDFFVLPVFNIDGYEYTWNR | | --- | | QSIGNTYEGR | | YDLAAHHSELMSVSQGAIAALR | | YIANHVLNNLY | | YNSWATINDWAISISSANPDLISR | | YTSGPGAATIYPAAGGSDDWAYDLGVK | | | |
| Acidic chitinase isoform C | | 52.5 | | ELLEAYEAEGK | | --- | | FTGHNSPLYQGSKDEGDLVYFNTDYAMR | | GTIDDGYEIAEIAK | | LTSSDTSVGAPASGPASAGTYTR | | NSEWVGFDTK | | TYGFDGMDLDWEYPGSR | | | |
| α-1-antitrypsin | | 47.8 | | ADFSGMTEEVK | | --- | | DAQHYYNSEAFGVDFSKPEIAAAEINK | | DLDADTVMMLINYMYFR | | LDDILMDMGMTDAFDYK | | NIFFSPVGISMALSLLAVGAK | | VDQDTTVQVDMMK | | VLHQAVMSVDEK | | | |
| Trypsin 4 | | 57.5 | | QSQDLPNPNEVSK | | --- | | SFNTWVQAGVVSFGK | | VSQYQNWISQYVR | | | |
| Elastase A | | 28.8 | | HDLSVNEEGSQTISAQK | | --- | | VSSFTDWVDK | | VSSFTDWVDKVMMNN | | WNSMFVALGNDIALIK | | | |
| Trypsin 5 | | 21.4 | | HDLSVNEEGSQTISAQK | | --- | | VSSFTDWVDK | | VSSFTDWVDKVMMNN | | WNSMFVALGNDIALIK | | | |
| Legumain-like protease | | 20.6 | | KQGIPDEQIVVMMYDDIANNPNNPFPGSIR | | --- | | MVIFMDSDNSQSVFK | | QGIPDEQIVVMMYDDIANNPNNPFPGSIR | | SVVDQTNVYK | | | |
| Trypsin 6 | | 28.8 | | EQYMTVSNVYIHPNWNR | | --- | | NNVAAGYDIALLR | | VVLGEHDIYK | | VVLGEHDIYKQEGR | | | |
|  | |  |  | | |
| Trypsin | 25.8 | | | HPSYNSNTLDNDVMLIK  LGEHNIDVTEGTEQFINSEK  LSSSAQINSYVK  TVSLPSSCASSGTSCLISGWGNMSASGSNYPS-R |  |
